# Disparities in naloxone prescriptions in a University Hospital during the COVID-19 pandemic

**DOI:** 10.1186/s12954-022-00667-9

**Published:** 2022-07-26

**Authors:** Kimberly Chieh, Ishika Patel, Lauren Walter, Li Li

**Affiliations:** 1 Department of Psychiatry and Behavioral Neurobiology, University of Alabama at Birmingham, 1720 University Blvd., Birmingham, AL 35294 USA; 2 Department of Emergency Medicine, University of Alabama at Birmingham, Birmingham, AL 35294 USA

**Keywords:** Naloxone, Opioid use disorder, Opioid overdose, Prevention, Health disparities

## Abstract

**Background:**

Per the CDC, it is estimated that 69,710 opioid overdose deaths occurred in the United States from September 2019 to September 2020. However, it is unclear whether naloxone prescribing also increased or otherwise fluctuated in this time. The objective of this study was to characterize the naloxone prescribing rate in patients with opioid use disorder (OUD) at the University of Alabama at Birmingham Hospital in 2019 and 2020.

**Methods:**

A cross-sectional, retrospective medical record review was performed on patients with OUD from January 2019 through December 2020. Naloxone prescribing, defined as either a written prescription or a provided take-home kit, was assessed for all patients with OUD.

**Results:**

In 2019, 11,959 visits were made by 2962 unique patients with OUD, compared to 11,661 visits from 2,641 unique patients in 2020; 609 naloxone prescriptions were provided in 2019 (5.1%) and 619 in 2020 (5.3%). In both years, most OUD-related visits and naloxone prescriptions were from and to male, white, individuals. Compared with 2019, more naloxone prescriptions were given to uninsured patients in 2020 (33.2% vs 44.3%, *p* < 0.05), and more OUD patients were admitted to inpatient settings (26.0% vs 31.2%, *p* < 0.05) and received more naloxone prescriptions in the inpatient setting (46.3% vs 62.0%, *p* < 0.05) in 2020. The proportion of frequent users (i.e., visits ≥ 4 times/year) increased in 2020 for the emergency department (21.5% vs 26.4%, *p* < 0.001) and inpatient setting (24.9% vs 28.6%, *p* = 0.03).

**Conclusions:**

Our findings indicate the need for improving naloxone awareness in providers and prescribing for patients with OUD, particularly in emergency department and outpatient settings. Our results also demonstrated a disparity in naloxone prescribing; a disproportionate number of opioid-related emergency department visits and overdose deaths were noted in Black people and frequent users.

## Background

From 1999 to 2019, there have been three waves of the opioid epidemic in the United States (U.S.) in which approximately 500,000 individuals total have died of opioid overdose [1]. The U.S. Food and Drug Administration (FDA) has approved three medications for opioid use disorder (MOUD): methadone, buprenorphine, and naltrexone. Buprenorphine can be administered in two forms: subcutaneous injections and sublingual, both of which are demonstrated to be effective in treating patients with OUD in both European and the U.S. [2–4]. Increasing evidence from Europe and the U.S. has demonstrated that opioid substitution treatment (OST) or MOUD are effective to reduce illicit opioid use and overdose fatalities [2, 5]. Despite this, in 2020, only 11.2% of people with OUD received MOUD treatment in the U.S, including the state of Alabama [6]. MOUD is often unavailable to those in need of it because of inadequate funding for treatment programs and a lack of qualified providers who can deliver these therapies.

Naloxone, is an opioid antagonist, which, if administered promptly following opioid ingestion, can reverse the opioid’s effect and prevent fatal overdose [7]. In hospital settings, naloxone has been utilized to quickly reverse an opioid overdose and to regulate the impact of opioids used in anesthesia [8]. An abundance of data supports take-home naloxone programs, in which individuals are trained to identify opioid overdoses and are equipped with naloxone for rescue resuscitation. These programs are associated with a decrease in mortality in those who misuse opioids [9]. From 2017 to 2018, the number of naloxone prescriptions nearly doubled from 270,000 in 2017 to 556,000 in 2018 in the United States [10]. However, despite these increases, for every 70 high-dose opioid prescriptions, only one naloxone prescription is provided [11]. This could signify potential areas for improvement in naloxone prescriptions and access. In addition, disparities have been noted in overdose treatment for certain demographic groups; Black people are the most at-risk population within the Southeastern region for having a greater number of hospital visits for nonfatal opioid overdose and overdose deaths [12].

The COVID-19 pandemic has resulted in further worsening of the opioid epidemic, including an escalation in nonfatal overdose events and deaths. The increase in overdose events is likely a result of several contributing factors, including disruption in naloxone distribution, social isolation, interruption in import of heroin and other drugs, increased fentanyl on the drug market, and decrease in access to substance use treatment. In March 2020, when the COVID-19 pandemic resulted in declaration of a national emergency, numbers of national nonfatal overdoses increased [13, 14]. In July 2020, numbers of suspected opioid overdoses peaked then gradually declined during subsequent months, but numbers were still higher in September 2020 than before the onset of the pandemic in March 2020 [15]. In contrast, there was a 26.3% decrease in individuals filling prescriptions for naloxone [16]. Furthermore, the rate of naloxone prescription-filling remained lower during the pandemic than the pre-pandemic time, especially for patients using Medicare insurance [16].

In Alabama, opioids have been involved in the majority of reported overdose deaths since 2015. In 2018, approximately half of the 775 overdose deaths reported in Alabama involved opioids and more than 6200 doses of naloxone were administered, a 35% increase from 2017 [17]. In 2020, drug overdoses in Alabama increased more than 20% from the year prior, making Alabama one of the 25 states with the highest overdose increase [18]. Furthermore, recent reports show that opioid-related overdose deaths have increased by 32.5% in just the first 6 months of 2020 in Jefferson County, the most populous county in Alabama [19]. The guidelines for providing OUD treatment in Alabama are overseen by the Alabama Department of Mental Health. Substance abuse treatment services include withdrawal management, residential treatment, intensive outpatient treatment, outpatient treatment, and/or MOUD. Naloxone is available to patients by prescription, but it can also be found at health departments, pharmacies, and naloxone training programs [20]. It is important to assess the distribution and prescription rates of naloxone to identify any opportunities for improvement to help prevent opioid overdose incidents and deaths in Alabama. Therefore, the objectives of this study were to determine: (1) naloxone prescription patterns for patients with OUD in the University of Alabama at Birmingham Hospital in 2019 and 2020; (2) association between patient demographics, including socioeconomic status, and naloxone prescribing; and (3) potential clinical implications for naloxone prescribing and access.

## Methods

This was a retrospective study approved by the Institutional Review Board at the University of Alabama at Birmingham (UAB). Informed consent was waived. Patients who presented and were diagnosed with OUD or were prescribed naloxone at the UAB Hospital from January 2019 to December 2020, were identified from electronic medical records (EMR). Naloxone prescribing is defined as either a written prescription or a provided take-home kit in this study given at UAB Hospital. At this institution, providers distribute naloxone at their discretion following specialty specific guidelines. EMR search was based on patients who were diagnosed with OUD per DSM-5 diagnostic criteria or were prescribed naloxone in the inpatient, outpatient, and emergency department settings. Two researchers (KC and IP) independently conducted EMR reviews to abstract patients’ data. Demographic characteristics, including sex, age, race/ethnicity, and registration verified insurance status, were also obtained from the EMR. We analyzed data from January through December of 2019 and 2020, respectively, and compared the number of total patients with OUD and naloxone prescriptions in these 2 years. Descriptive statistical and Chi-square analyses were performed using SPSS version 27 (IBM) and JMP 14 (SAS Institute), respectively.

## Results

In 2019, there were 11,959 visits from 2962 unique patients with OUD, and 609 naloxone prescriptions were provided for these patients. In 2020, there were 11,661 visits from 2641 unique patients with OUD, and 619 naloxone prescriptions were provided for these patients. The average age for patients presenting with OUD was 43.4 ± 14.0 in 2019 and 42.3 ± 13.5 in 2020. The average age of patients who received a naloxone prescription was 44.4 ± 14.7 in 2019 and 41.9 ± 12.6 in 2020 (Table 1).

**Table 1:** Characteristics of patients

	Number of naloxone prescriptions		*p-*value	Number of patients with OUD		*p*-value
Year	2019	2020		2019	2020	
Total	609	619		2962	2641	
*Age (years)*						
Mean (SD)	44.4 ± 14.7^a^	41.9 ± 12.6^a^	^a^	43.4 ± 14.0	42.3 ± 13.5	^a^
*Sex*						
Female	300 (49.3%)	277 (44.7%)	0.11	1461 (49.3%)	1272 (48.2%)	0.39
Male	309 (50.7%)	342 (55.3%)		1501 (50.7%)	1369 (51.8%)	
*Race*						
Black	123 (20.2%)	142 (22.9%)	0.49	651 (22.0%)	527 (20.0%)	0.17
White	472 (77.5%)	462 (74.6%)		2223 (82.58%)	2030 (76.9%)	
Other^b^	14 (2.3%)	15 (0.81%)		88 (3.0%)	84 (3.2%)	
*Ethnicity*^c^						
Non-Hispanic	575 (94.4%)	587 (94.8%)		2829 (95.5%)	2492 (94.4%)	
Hispanic	3 (0.5%)	2 (0.3%)		13 (0.4%)	16 (0.6%)	
Unknown	31 (5.1%)	30 (4.8%)		120 (4.1%)	133 (5.0%)	
*Insurance*						
Public	295 (48.4%)	251 (40.5%)	** < 0.05**	1101 (37.2%)	943 (35.7%)	0.23
Private	112 (18.4%)	94 (15.2%)		761 (25.7%)	659 (25.0%)	
Uninsured	202 (33.2%)	274 (44.3%)		1100 (37.1%)	1039 (39.3%)	
*Encounter*						
Emergency	118 (19.4%)	117 (18.9%)	** < 0.05**	1123 (37.9%)	899 (34.0%)	** < 0.05**
Inpatient	282 (46.3%)	384 (62.0%)		770 (26.0%)	824 (31.2%)	
Outpatient	209 (34.3%)	118 (19.1%)		1069 (36.1%)	918 (34.8%)	

Bold: Most naloxone prescriptions were provided for individuals on public insurance or without insurance (self-pay) in 2019; however, there was a significant change between 2019 and 2020, where the number of uninsured patients receiving naloxone prescriptions increased (202 vs 274, *p* < 0.05). Compared with 2019, more patients were admitted to the inpatient setting in 2020 (770 vs 824, *p* < 0.05), and more patients in the inpatient setting received naloxone prescriptions in 2020 (282 vs 384, *p* < 0.05)

*OUD* opioid use disorder. Data are presented in number and percentage, *N* (%), except ages. SD indicates standard deviation

^a^Excluded from Chi-square analysis due to missing age data for deceased patients (84 in patients with OUD group, 26 in naloxone prescriptions group)

^b^Includes Asian, Hispanic, and unknown

^c^Excluded from Chi-square analysis due to low numbers of Hispanic cohort

In both years, more males than females presented to the hospital with OUD and received naloxone prescriptions, although no statistically significant differences were noted between years in this regard. White patients had the highest volume of visits and received the most naloxone prescriptions. Black patients had the second highest volume of visits and naloxone prescriptions.

Insurance status was similar among patients with OUD seeking medical care between 2019 and 2020. Most naloxone prescriptions were provided for individuals on public insurance or without insurance (self-pay) in 2019; however, there was a significant change between 2019 and 2020, where the number of uninsured patients receiving naloxone prescriptions increased (202 vs 274, *p* < 0.05). Compared with 2019, more patients were admitted to the inpatient setting in 2020 (770 vs 824, *p* < 0.05), and more patients in the inpatient setting received naloxone prescriptions in 2020 (282 vs 384, *p* < 0.05).

Secondary analysis of sex distribution in regards to race and insurance did not display significant changes in either race or sex over a 2-year period or any significant racial or sex-based discrepancies in naloxone prescriptions as compared to visits from patients with OUD.

A breakdown of emergency, inpatient, and outpatient visit numbers by patients with OUD is displayed in Table 2. Compared with 2019, the proportion of frequent users, defined as having four or more visits in a year [21], to the emergency department was greater in 2020 (26.4% vs 21.5%, *p* < 0.001). Regarding inpatient visits, fewer patients were admitted a single time in 2020 than 2019 (293 vs 324), but there was an increase in the number of patients who were admitted more than once (*p* = 0.03). There was a significant decrease between 2019 and 2020 for all patient presentation frequencies in the outpatient setting (*p* = 0.0487).

**Table 2:** Number of presentations by persons with OUD to medical settings, 2019 and 2020

	2019	2020	*p*-value
*Emergency*			
Single presentation	439 (39.1%)	263 (29.3%)	< 0.001
2–3 presentations	442 (39.4%)	399 (44.4%)	
≥ 4 presentations	242 (21.5%)	237 (26.4%)	
*Inpatient*			
Single presentation	324 (42.1%)	293 (35.6%)	0.03
2–3 presentations	254 (33.0%)	295 (35.8%)	
≥ 4 presentations	192 (24.9%)	236 (28.6%)	
*Outpatient*			
Single presentation	261 (24.4%)	186 (20.3%)	0.0487
2–3 presentations	363 (34.0%)	309 (33.7%)	
≥ 4 presentations	445 (41.6%)	423 (46.1%)	

Data are presented in number and percentage, *N* (%)

## Discussion

Overall, this study demonstrates a significant gap in naloxone prescriptions when compared to the number of visits from patients with OUD. In addition, these results highlight the changes in naloxone prescribing among various encounter settings, as well as changes in OUD visits and patient types, during the COVID-19 pandemic in a tertiary medical center. There was a significant increase from 2019 to 2020 in inpatient visits from patients with OUD and in naloxone prescriptions provided in the inpatient setting. This difference could be due to closures of treatment facilities and clinics for patients with OUD during the COVID-19 pandemic, resulting in increased inpatient hospital admissions for OUD patients [16, 22]. In addition, between 2019 and 2020, the proportion of repeat visits increased. In light of many treatment facility closures due to COVID-19, this increased presentation frequency to the emergency department by persons with OUD could indicate a lack of access to substance use disorder treatment facilities or mental health resources that may have been available before the pandemic, leading to subsequent utilization of the emergency department as a ‘safety net.’ Similarly, more patients were admitted to the inpatient setting multiple times in 2020 compared with 2019, indicating the potential exacerbation and increased severity of opioid-related medical issues caused by the COVID-19 pandemic [22]. Outpatient visits decreased across all presentation frequencies from 2019 to 2020, consistent with national data [23]. The UAB Hospital adopted telemedicine after the onset of the pandemic to accommodate patient needs, but outpatient visits still remained lower than pre-pandemic levels. Our data again highlight that the COVID pandemic changed patients’ health visit-related behaviors, which are similar to COVID-19-related changes in substance use and outcomes reported by others [24–26].

Compared with inpatient settings, our data indicate that the naloxone prescribing rate was lower in both emergency department and outpatient settings. Prescribing pattern differences in clinical settings could be due to the inpatient setting being afforded more time for patient care teams to address the OUD diagnosis and provide substance use counseling, naloxone training, and prescription. Emergency department and outpatient barriers reported by other health systems include time, stigma or lack of knowledge surrounding naloxone, medication cost for patients and the hospital, and administrative logistics when attempting to prescribe naloxone to patients [23, 24]. Additionally, a patient with OUD who presents to the emergency department or outpatient setting with a non-opioid related medical concern (e.g., bronchitis), may not be prescribed naloxone; OUD may represent a non-acute issue which is unsuitable to be addressed within the confines of a limited clinical setting. Furthermore, some patients with a documented diagnosis of OUD may not actively need naloxone, if they are in successful recovery for example, thereby contributing to the low naloxone prescription to OUD diagnosis ratio. In some instances, however, the emergency department and outpatient setting naloxone prescription gap may represent a critical mismatch which warrants intervention. Opportunities to address this include reflex co-prescription or automated flags to suggest prescription of naloxone with full opioid agonists for high-risk patients, particularly those with known history of OUD, and improved broad educational efforts to advertise the availability of and instructional use for naloxone, targeting OUD patients as well as providers.

Regarding prescription differences across insurance statuses, significantly more uninsured patients received naloxone in 2020 compared to 2019, and a relative decrease in publicly and privately insured patients receiving naloxone was also observed in the same time frame. This could be a potential indicator of the socioeconomic impacts of the COVID-19 pandemic on patients, like job loss, resulting in an increase in the uninsured cohort. Isolation measures and travel restrictions put in place at the onset of the pandemic caused all economic sectors to have a reduced workforce which undoubtedly impacted individual access to private insurance [27]. This may have had further influence on patient access to naloxone prescriptions, as uninsured patients often have difficulties establishing connections with physicians or utilizing healthcare resources [28]. Of additional note however, national data extracted from retail pharmacies around the country indicate that even patients who retained public insurance, specifically Medicaid, faced increased difficulty in accessing naloxone after the onset of the COVID-19 pandemic [16].

Access to naloxone has also been shown to be related to race and socioeconomic factors including income and employment [29]. Our results also demonstrated the presence of racial disparities as fewer Black people received naloxone prescriptions than whites. Despite being historically and stereotypically considered a disease impacting majority white, male patients in the United States, recent demographic data of people with OUD have shifted to include increasing percentages of women and racial minorities [12, 30]. Another study also showed opioid-related deaths among Black people rose 38% from 2018 to 2019 while rates for other racial groups did not significantly change [31]. At our own institution, this disparity was further emphasized during the height of the COVID-19 pandemic when a disproportionate number of opioid-related emergency department visits and overdose deaths were noted in Black people [12]. Though the data from this study did not demonstrate any sex-based differences or discrepancies, these national trends are certainly of interest for further research.

Our data also suggest additional social inequities beyond race, as more people of lower socioeconomic status, determined by their insurance status as a proxy, were diagnosed with OUD. Opioid visits and overdose deaths have previously been associated with socioeconomic stressors; Hollingsworth, et al. demonstrated that as county unemployment rate increased by one percentage point, opioid death rate per 100,000 rose by 3.6% and the opioid overdose emergency department visit rate increased by 7.0% [32]. Consistent with others’ reports, our findings indicate that an individual’s race and socioeconomic status may place them at a disadvantage when it comes to OUD and naloxone. These concerning trends highlight the need for a racially and socially inclusive approach to the opioid epidemic, particularly for those racial and socioeconomic groups which are already severely impacted by healthcare inequities.

Strengths of this study include the use of DSM-5 diagnostic codes in the EMR search, a medium sample size, and a focus on Alabama, one of the states with the highest overdose increases. There are several limitations to this study. The first 3 months of 2020 were included in the comparison between years as an indicator of COVID-related changes, but significant disruptions in care due to COVID-19 may not have occurred until mid-March or April of 2020. These 3 months may confound the data but should not change the overall data trends. This study was conducted over a limited time period with data extracted from a single site and as such, results may not be generalizable to other timeframes or locations. Data collection from other geographical regions may provide better insight in naloxone prescription patterns. Also, naloxone prescription numbers within our study are not indicative of the number of prescriptions filled by patients, only the number of written prescriptions and no cost kits distributed by healthcare providers in the hospital. More in-depth research is needed to understand prescription-filling patterns and trends in demographic usage of naloxone.

## Conclusion

Our findings demonstrate low prescription rates which suggest that naloxone is not being fully utilized for patients with OUD in the clinical setting. In addition, this study demonstrates a disparity in naloxone prescribing; a disproportionate number of opioid-related emergency department visits and overdose deaths were noted in Black people and frequent users, further underscoring preexisting racial and socioeconomic inequities. Finally, our study illustrates the urgent need to expand naloxone access for high-risk populations in a multitude of clinical settings. Opportunities exist for OUD clinical practice improvement, as it relates specifically to naloxone prescribing, and future advocacy efforts should focus on inclusion of these interventions, particularly for at-risk and vulnerable populations.

## Data Availability

The datasets used and/or analyzed during the current study are available from the corresponding author on reasonable request.

## Abbreviations

CDC: Centers for Disease Control and Prevention
DSM-5: Diagnostic and Statistical Manual of Mental Disorders
EMR: Electronic medical record
FDA: Food and Drug Administration
MOUD: Medication for opioid use disorder
OST: Opioid substitution treatment
OUD: Opioid use disorder
SD: Standard deviation
UAB: University of Alabama at Birmingham
U.S.: United States
